# The eyes have it

**DOI:** 10.7554/eLife.24896

**Published:** 2017-02-06

**Authors:** Mehmet Keleş, Mark A Frye

**Affiliations:** Department of Integrative Biology and Physiology, University of California, Los Angeles, Los Angeles, United States; Department of Integrative Biology and Physiology, University of California, Los Angeles, Los Angeles, United Statesfrye@ucla.edu

**Keywords:** visual behavior, lobula, feature detection, loom detection and avoidance behaviors, optic glomeruli, retinotopy, *D. melanogaster*

## Abstract

Molecular genetic experiments are revealing how the fly brain generates behavioral responses to visual stimuli.

**Related research article** Wu M, Nern A, Williamson WR, Morimoto MM, Reiser MB, Card GM, Rubin GM. 2016. Visual projection neurons in the *Drosophila* lobula link feature detection to distinct behavioral programs. *eLife* **5**:e21022. doi: 10.7554/eLife.21022

Like us, flies depend on their sense of sight. When a fly perceives an approaching object, such as a fly swatter, it repositions itself and executes an escape strategy in less time than the blink of an eye (Card and Dickinson, 2008). Flies produce an impressive repertoire of visual behaviors, including escape, with a brain that contains a relatively small number of neurons. *Drosophila melanogaster*, the fruit fly, has become an enormously useful model for studying visual behavior, yet the neural mechanisms for transforming object signals (such as an approaching swatter) into motor actions (escape) remain poorly understood.

The *Drosophila* retina is composed of roughly 700 hexagonal facets, each viewing a small portion of the visual field, and signals from the photoreceptors within each facet are processed by four optic lobes in the brain. The processing in these optic lobes happens in a retinotopic fashion: in other words, signals from neighboring facets are passed through the optic lobes by neighboring columns of neurons. The signals are first processed by an optic lobe called the lamina, followed by the medulla, and then the lobula and the lobula plate (Figure 1). The last two lobes collate retinotopic information from all the inputs and project axons that carry filtered signals to structures elsewhere in the brain.

**Figure 1.**
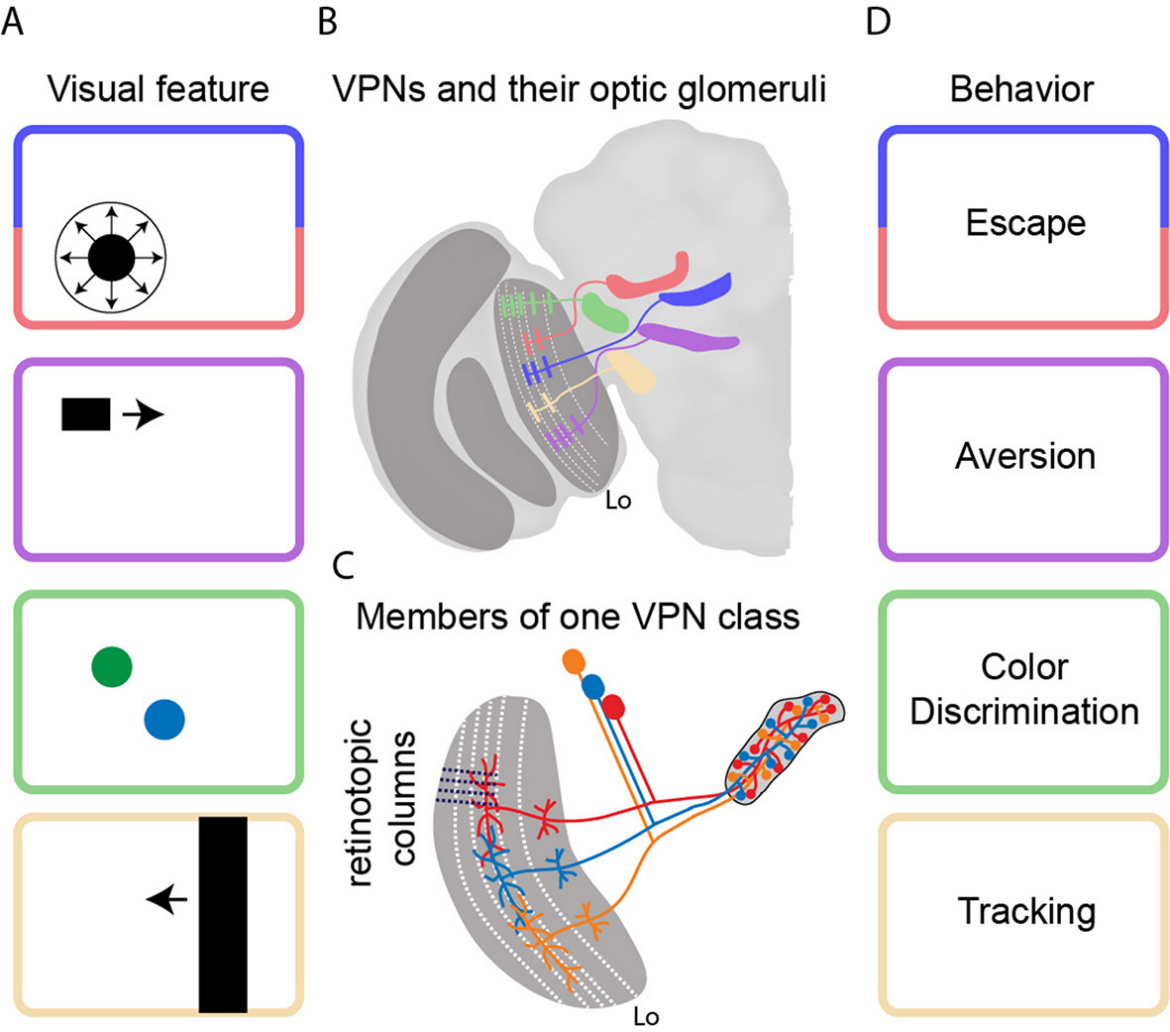
Transforming visual signals into motor actions. (**A**) Visual features that are important to the fly include looming (top), small moving objects, colors, and vertical edges. (**B**) Wu et al. identified 22 different classes of visual projection neurons (VPNs) in the lobula (Lo), with all the neurons in a given class projecting to a specific glomerulus in the brain. Five examples are shown schematically. Wu et al. also observed that the neurons have dendritic innervations within anatomically distinct layers of the lobula (indicated by white dashed lines). (**C**) Neighboring columns of neurons in the lobula (indicated by black dashed lines) sample neighboring regions of space. The neurons in a given VPN class have overlapping dendritic fields, which corresponds to overlaps in the sampling of visual space. The axon terminals, on the other hand, completely innervate the glomerulus for that VPN class. (**D**) It is thought that each VPN class responds to a visual feature (panel A) and contributes to a particular form of behavior (panel D).

Lobula plate neurons have been studied for 60 years and it is known that they compute patterns of visual motion across the eye to guide navigation tasks (Borst, 2014). However, much less is known about the lobula, even though it contains four times as many neurons (Strausfeld, 1976). Now, in eLife, Aljoscha Nern, Gerald Rubin and colleagues at the Janelia Research Campus – including Ming Wu and Nern as joint first authors – report the results of a series of anatomical and behavioral experiments to understand the architecture and functions of these neurons (Wu et al., 2016). In particular they identify 22 different classes of visual projection neurons (VPN) in the lobula, and show that specific classes of neurons elicit specific visual behaviors, such as escape.

The power of *Drosophila* genetics is deployed in full force here. Wu et al. first screened large collections of genetically modified flies to find lines in which it is possible to fluorescently label all the retinotopic neurons of a given VPN class that project from the lobula to the center of the brain. Then they stochastically labeled a few individual neurons in each of the 22 VPN classes with different fluorescent colors. This systematic approach allowed them to take high-resolution pictures of input dendrites and output axon terminals, and to demonstrate that each VPN class had a characteristic number of cells, dendritic span, and axon output location (Figure 1B). Whereas the input dendrites in each class were organized in a retinotopic fashion, the axon terminals were fully intermingled to form an optic glomerulus. Strikingly, it would appear that the spatial information contained in the inputs is thrown away because it is not contained in the outputs (Figure 1C).

Next, Wu et. al. investigated the behavioral role of each VPN class by testing whether the use of light to activate the neurons in a particular class provoked any observable behavioral reactions. Activation of two classes (called LC6 and LC16) resulted in significant jumping and backward walking, which are hallmarks of visual escape behavior. In further tests strong calcium currents were detected in both classes when the flies were presented with a looming stimulus (like an approaching fly swatter). It would appear that LC6 and LC16 neurons transform looming visual information into the motor control of a rapid escape behavior (also see von Reyn et al., 2014).

In addition to shedding new light on lobula projection neurons, the work of Wu et al. also raises exciting new questions. 1) What is the functional benefit of losing the retinotopic information that was contained in the input to the lobula? 2) Individual members of a given class have overlapping dendritic fields, which means that a given region of visual space is covered more than once: what is the benefit to this oversampling? 3) As Wu et al. demonstrate, a single type of behavior can be initiated by more than one class of neurons. This means that activating a given class may be sufficient to provoke a specific behavior, but silencing the same class does not necessarily quell that behavior. What gives rise to the apparent redundancy within the brain? 4) We recently performed a complimentary comprehensive physiological characterization of one these VPN classes: this study revealed complex spatial inhibitory interactions, indicating that only a fraction of the neurons in this class are activated by the salient visual stimulus (Keleş and Frye, 2017). Therefore, as Wu et al. note, the use of optogenetic techniques to simultaneously activate the whole population of neurons does not mimic what happens naturally. How does the output sent to the glomerulus reflect the spatial dynamics of the inputs?

Based on what we currently know about the functional properties of lobula visual projection neurons (Keleş and Frye, 2017; Mu et al., 2012), activity within a given optic glomerulus seems to correspond to the presence of a visual feature rather than its direction of movement or spatial location. In flies and mammals, the spatial pattern of olfactory glomeruli can signal the identity and intensity of an odorant (Wang et al., 2003). Perhaps something similar is happening here, with the pattern of activation across different optic glomeruli signaling particular features of visual objects rather than their motion or location. The approaches developed by Wu et al. are likely to prove very useful for exploring this hypothesis and for studying how visual representations are transformed into behavioral commands more generally.
